# Perspectives and evaluation on the effect of financial burden relief of medical insurance for people with catastrophic diseases and its influencing factors

**DOI:** 10.3389/fpubh.2023.1123023

**Published:** 2023-04-06

**Authors:** Pengfei Guo, Yinghua Qin, Rizhen Wang, Jiacheng Li, Jingjing Liu, Kexin Wang, Ye Li, Zheng Kang, Yanhua Hao, Huan Liu, Hong Sun, Yu Cui, Linghan Shan, Qunhong Wu

**Affiliations:** ^1^Department of Social Medicine, School of Public Health, Harbin Medical University, Harbin, Heilongjiang, China; ^2^Department of Health Economy and Social Security, College of Humanities and Management, Guilin Medical University, Guilin, China

**Keywords:** medical insurance, people with catastrophic diseases, financial burden, influencing factors, China

## Abstract

**Background:**

Catastrophic disease sufferers face a heavy financial burden and are more likely to fall victim to the “illness-poverty-illness” cycle. Deeper reform of the medical insurance system is urgently required to alleviate the financial burden of individuals with catastrophic diseases.

**Methods:**

Data were obtained from a cross-sectional questionnaire survey conducted in Heilongjiang in 2021, and logistic regression and restricted cubic spline model was used to predict the core factors related to medical insurance that alleviate the financial burden of people with catastrophic diseases.

**Results:**

Overall, 997 (50.92%) medical insurance-related professionals negatively viewed financial burden relief for people with catastrophic diseases. Factors influencing its effectiveness in relieving the financial burden were: whether or not effective control of omissions from medical insurance coverage (OR = 4.04), fund supervision (OR = 2.47) and degree of participation of stakeholders (OR = 1.91). Besides, the reimbursement standards and the regional and population benefit package gap also played a role. The likelihood of financial burden relief increased by 21 percentage points for each unit increase in the level of stakeholder discourse power in reform.

**Conclusion:**

China’s current medical insurance policies have not yet fully addressed the needs of vulnerable populations, especially the need to reduce their financial burden continuously. Future reform should focus on addressing core issues by reducing the uninsured, enhancing the width and depth of medical insurance coverage, improving the level and capacity of medical insurance governance that provides more discourse power for the vulnerable population, and building a more responsive and participatory medical insurance governance system.

## Introduction

There is a kind of social disease in the world whose name called the disease of poverty. Poverty is one of the major obstacles to stable and sustainable social development. The number of people living in extreme poverty (with an income of less than $1.90 per day) went from 36% in 1990 to 10% in 2015, 9.2% in 2017, 8.4% in 2019, and 9.1–9.4% in 2020 (1, 2). It shows that the pace of global poverty eradication has slowed recently. A number of factors are to blame, but the COVID-19 pandemic may be particularly responsible for reversing decades of progress. One thing seems to have stayed the same - expensive health care continues to impoverish people. A survey of 89 countries by the World Health Organization (WHO) found that the proportion of people suffering severe financial hardship because of diseases was growing at an annual rate of 11 per cent (3). Among a large number of sick people, the most vulnerable are those with catastrophic diseases, who are more likely to suffer severe financial burdens due to prolonged, expensive treatment and regular drug maintenance. It was estimated that 150 million people experience catastrophic health expenditure (CHE) each year due to healthcare costs (4). In Burkina Faso, an increase by one illness among adults significantly increased the probability of CHE from 1.5 to 1.7 times (5). The proportion of households experiencing CHE in India increased between 1993 and 2014, especially among households with older adults (6). A subgroup of households in sub-Saharan Africa may encounter CHE when utilizing medical services, with the incidence rising and generally being greater between 2010 and 2020 (7). A national survey shows that the percentage of Malaysian households suffering from CHE is 2.8% and increasing (8). In China, disease is the main cause of people’s poverty (9, 10). According to the statistical bulletin released by China’s National Bureau of Statistics, among the causes of poverty in rural China, poverty caused by disease accounts for 44.1%, involving nearly 20 million people, including 7.34 million people suffering from catastrophic and chronic diseases (11).

The financial burden of catastrophic diseases has become a pressing issue for the global medical insurance system to address. Medical insurance acts as a third-party payer between patients and providers to tackle the high cost of healthcare. It is an essential mechanism for managing health risks and addressing the risk of financial loss. Several studies have empirically tested the potential burden-reducing effects of medical insurance. Among them, Medicaid in the United States has lifted at least 2.6 million people out of poverty by subsidizing direct medical costs and reducing out-of-pocket medical costs from $871 to $376 per beneficiary (12). Actually, the burden-reducing effect of medical insurance has been positively demonstrated in countries such as China, South Korea, India, and Ghana (13–16). However, some studies have argued against the effectiveness of medical insurance in reducing the burden (9), especially for socially disadvantaged groups (17), such as the older people, the very sick, and the rural population (18–20). In addition, as shown in an empirical study of the PMJAY, a national publicly funded medical insurance program in India, and the incidence of out-of-pocket, CHE, PMJAY did not reduce the financial burden of access to quality medical services for the poor and vulnerable (21). A study conducted in China also found that medical insurance plans did not actually reduce the risk of catastrophic medical expenditure, with the highest rates of CHE and poverty due to cancer, chronic respiratory diseases, and diabetes (22). The picture is also bleak for rare diseases, with medical and drug expenditures for rare disease patients in Taiwan, China increasing dramatically over the past decade. In 2014, drug expenditures accounted for 88.75% of rare diseases’ medical expenditures. With the development of rare disease drugs and their newer iterations, drug expenditures were likely to increase further (23). All of the above studies showed the failure of medical insurance to alleviate the financial burden of vulnerable populations such as those with catastrophic diseases. Scholars had different opinions on this. Some considered that the rapid increase in medical care costs has undermined the true effectiveness of insurance (24); others argued that vulnerable populations such as those with chronic diseases, have an increasing demand for medical services and that these groups are at constant risk of financial hardship and poor health in the absence of effective protection mechanisms (25, 26).

This study built on the above research by focusing on the impact and effect of medical insurance system design, problems, and reform measures on the financial burden of people with catastrophic diseases. Most current research on medical insurance and financial burden still focused on the impact of the presence or absence of medical insurance and the type of insurance on the overall population burden. There were also studies of the impact of individual medical insurance on the burden of disease in specific populations (22, 27–29). Several studies analyzed the benefits of medical insurance integration measures in terms of enrollee satisfaction and CHE (30–32). Although a great deal of research has been done on medical insurance and the burden of diseases, there is a little in-depth exploration into the medical insurance system itself. Questions such as how medical insurance can focus on key issues and implement effective reforms to reduce the financial burden of people with catastrophic diseases still need to be answered. In addition, it is necessary to understand the views of medical insurance-related professionals on the relationship between medical insurance policies and the financial burden of people with catastrophic diseases. They are the architects and implementers of medical insurance policies. This study drew on the experience of a large number of medical insurance-related professionals. It aimed to understand how medical insurance-related professionals perceive the effectiveness and weaknesses of China’s medical insurance in alleviating the financial burden of people with catastrophic diseases. Also, to quantify the related medical insurance issues and governance measures and explore the determinants that may affect the effectiveness and provide reference evidence for deepening the reform of the medical insurance system and sustaining the promotion of medical insurance poverty alleviation.

## Methods

### Data source and sampling method

A cross-sectional questionnaire survey was conducted in 2021. Respondents were selected by multi-stage stratified cluster random sampling. We conducted the first-stage sampling of 12 cities, including Harbin, Qiqihar, and Daqing, in Heilongjiang Province. The second-stage sampling was conducted at the institutional level by cluster sampling of staff in medical insurance administrative agencies, other relevant departments, medical institutions and third-party institutions such as universities and research institutes. All staff members included were invited to participate in the survey, such as medical insurance administrators, handling staff, accounting staff of medical institutions, and university experts and scholars engaged in medical insurance-related departments, for the questionnaire survey. Due to the sporadic outbreak of COVID-19 and the dynamic zero-COVID policy adopted in China, this survey was conducted online, using the Questionnaire Star platform to upload and disseminate the questionnaire. The entire survey lasts approximately 65 days from January to March 2021. To ensure the quality of the data, we asked the respondents to read a questionnaire introduction note before completing the questionnaire, when respondents complete the electronic questionnaire, four questionnaire auditors from Harbin Medical University perform quality control. They excluded questionnaires that did not meet the study as well as quality requirements. Specific exclusion criteria: (1) Questionnaire completion time less than 500 s. (2) Age less than 16 years. (3) Greater consistency in the choice of answers to questionnaire entries. (4) The IP belonged to a place other than Heilongjiang. (5) There are other situations that violate the normal answer logic. A total of 2054 respondents completed the questionnaire, after review, those unqualified questionnaires were excluded. The final sample size we included in the analysis was 1958.

## Variables

### Dependent variable

Distinct from conventional studies of the financial burden of diseases, our survey did not apply the widely used objective indicators such as CHE, impoverishing medical expenditures (IME), and out-of-pocket expenses (OOPE). It must be acknowledged that these indicators fail to account for indirect costs like lost wages due to missed work or transport for medical appointments, as well as the intangible costs associated with a person’s lower quality of life due to illness, unmeasured emotional stress, and shortened lifespan. We believe that subjective evaluations by medical insurance-related professionals based on a policy evaluation may provide a different perspective on disease burden research. The dependent variable of this paper was the evaluation of medical insurance-related professionals on the medical insurance’s effectiveness in financial burden alleviation for people with catastrophic diseases. The response options were based on a five-point Likert scale (1 = no relief, 2 = slight relief, 3 = average relief, 4 = larger relief, 5 = very large relief). To comply with the modeling requirements of logistic regression, we divided the respondents’ choices into two categories: unrelieved = 0 (including no relief, slight relief, and average relief); and relieved =1 (including larger relief and very large relief).

### Independent variables

We drew on the independent variables addressed in previous relevant studies (10, 18, 30, 33) and further consulted with medical insurance experts to avoid omitting important variables. The main independent variables of this paper: (1) existing design problems of the medical insurance scheme that may influence the alleviation effect (including medical insurance reimbursement standards for patients, regional and population differences in benefit package of medical insurance due to the different levels of economic development, etc.); (2) the management issues that may lead to the fund waste of medical insurance unchecked and therefore affect its alleviation ability (including payment method’s capacity to control the cost of medical insurance, medical insurance fund supervision, leakage control, etc.); (3) the respondents were asked to evaluate the extent to which medical insurance governance measures were achieved (including the level of participation and voice of medical insurance stakeholders in reform and governance, etc.).

### Control variables

We controlled the confounding effects of respondents’ demographic and socioeconomic characteristics (age, gender, education, etc.).

### Statistical analysis

The data were analyzed using STATA 16.0 and R 4.1.3. Statistical descriptions (percentages and frequency) were used to describe the sample. We conducted the Chi-square and Mann–Whitney U test/t-test to determine the relationship between each variable and the evaluation of medical insurance-related professionals on the effectiveness of financial burden alleviation for people with catastrophic diseases. We then constructed a model that included the variables that showed significance in the univariate analysis (α = 0.05). Immediately after, we included variables that did not show a relationship in the univariate analysis but were considered to be related by medical insurance experts in the model for consideration. For example, although the variable “capacity to control the cost of medical insurance payment methods “did not show any association, it was included in the regression model for further testing and analysis as suggested by medical insurance experts. We also used restricted cubic splines to flexibly model the relationship between the variable “level of stakeholders’ involvement in the process of medical insurance reform and governance” and the evaluation of medical insurance-related professionals on the effectiveness of financial burden alleviation for people with catastrophic diseases. The system automatically generated reference values and the four knots were used to draw the restricted cubic spline.

## Results

### Basic information of the interviewee

The overall characteristics of each respondent were shown in Table 1. They were mostly female (67.67%) and between the ages of 31 and 48. The majority (62.76%) held a bachelor’s degree or higher. Additionally, 77.12% of the respondents had positions in divisions that dealt with medical insurance. We surveyed their opinions on the issues with medical insurance, the efficiency of the measures, and its management. In the opinion of 53.37% of the respondents, patient medical insurance reimbursement requirements are still woefully inadequate to help patients fight catastrophic diseases. The majority of respondents (62.41%) said that the supervision of medical insurance funds was efficient. However, 69.92% of the respondents believed that there was insufficient stakeholder participation in the various medical insurance reforms.

**Table 1:** Respondents’ basic characteristics and univariate analysis.

Variables	Total sample (*n* = 1958)	Unrelieved (*n* = 997)	Relieved (*n* = 961)	*χ*^2^/*Z*/*t*
Medical insurance reimbursement standards				40.11^***^
High	913(46.63)	395 (39.62)	518 (53.90)	
Low	1,045 (53.37)	602 (60.38)	443 (46.10)	
Regional and population benefit package gap of medical insurance scheme				4.15^*^
Unserious	1,079 (55.11)	527 (52.86)	552 (57.44)	
Serious	879 (44.89)	470 (47.14)	409 (42.56)	
Level of stakeholders’ discourse in medical insurance reform				−13.46^***^
Scoring 1–10	8 (6,9)	7 (5,8)	8 (7,10)	
Fragmentation of the medical insurance system				20.60^***^
No	814 (41.57)	365 (36.61)	449 (46.72)	
Yes	1,144 (58.43)	632 (63.39)	512 (53.28)	
Ability of vulnerable groups to participate in the insurance system				14.63^***^
Sufficient	957 (48.88)	445 (44.63)	512 (53.28)	
Insufficient	1,001 (51.12)	552 (55.37)	449 (46.72)	
Capacity to control the cost of medical insurance by current payment methods				1.83
Sufficient	1,378 (70.38)	688 (69.01)	690 (71.80)	
Insufficient	580 (29.62)	309 (30.99)	271 (28.20)	
Degree of participation of medical insurance stakeholders in the reform				115.26^***^
Low	1,369 (69.92)	806 (80.84)	563 (58.58)	
High	589 (30.08)	191 (19.16)	398 (41.42)	
Medical insurance fund supervision				366.92^***^
Ineffective	736 (37.59)	580 (58.17)	156 (16.23)	
Effective	1,222 (62.41)	417 (41.83)	805 (83.77)	
Control of omissions from medical insurance coverage				448.58^***^
Ineffective	797 (40.70)	636 (63.79)	161 (16.75)	
Effective	1,161 (59.30)	361 (36.21)	800 (83.25)	
Management of online charity fundraising platforms				3.96^*^
Ineffective	1,031 (52.66)	503 (50.45)	528 (54.94)	
Effective	927 (47.34)	494 (49.55)	433 (45.06)	
Gender				0.13
Male	633 (32.33)	326 (32.70)	307 (31.95)	
Female	1,325 (67.67)	671 (67.30)	654 (68.05)	
Age				3.07^**^
≥16	39.97 ± 8.85	40.57 ± 8.90	39.35 ± 8.75	
Education level				14.29^**^
Junior college and below	729 (37.23)	347 (34.80)	382 (39.75)	
Bachelor	1,104 (56.38)	568 (56.97)	536 (55.78)	
Master	107 (5.46)	71 (7.12)	36 (3.75)	
PhD and above	18 (0.92)	11 (1.10)	7 (0.73)	
Type of work unit				61.54^***^
Medical insurance administrative agencies	1,510 (77.12)	701 (70.31)	809 (84.18)	
Other related departments	142 (7.25)	82 (8.22)	60 (6.24)	
Medical institutions	115 (5.87)	87 (8.73)	28 (2.91)	
Third-party institutions	191 (9.75)	127 (12.74)	64 (6.66)	

^***^p < 0.001, ^**^p < 0.01, ^*^p < 0.05.

### Analysis of differences

As shown in Table 1, more than half (50.92%) of the respondents evaluated the effect of alleviating the financial burden of people with catastrophic diseases as unsatisfactory. Univariate statistical analysis showed that medical insurance reimbursement standards, regional and population benefit package differences of medical insurance were factors that influence the evaluation of medical insurance-related professionals, besides, the level of the stakeholders’ involvement in medical insurance and its governance reform, fragmentation of the medical insurance system, and the ability of vulnerable groups to participate in the insurance system also play a role. Respondents who perceived a high degree of stakeholders’ participation in the reform of medical insurance and the effectiveness of supervision of medical insurance funds tended to believe that the degree of financial burden relief for the catastrophic disease population was better. Those who showed negative perceptions about the management of online charity fundraising platforms were more likely to disagree that the financial burden was alleviated.

### Logistic regression model

Further exploring the effects of the above-mentioned factors on their impact on the degree of financial burden relief for people with catastrophic diseases, a multiple logistic regression analysis was conducted with seven significant predictors (*p* < 0.05) identified. Including (1) the medical insurance reimbursement standards (OR = 0.60). (2) The regional and population benefit package gap of medical insurance scheme (OR = 0.78). (3) The level of stakeholders’ discourse in medical insurance reform (OR = 1.21). (4) The ability of vulnerable groups to participate in the insurance system (OR = 0.79). (5) The degree of participation of medical insurance stakeholders in the reform (OR = 1.91). (6) The effectiveness of medical insurance fund supervision (OR = 2.47). (7) The effectiveness of the control of omissions from medical insurance coverage (OR = 4.04). For details, see Table 2.

**Table 2:** Logistic regression analysis.

Variables	β	SE	Wald	OR(95% *CI*)	*p*
Medical insurance reimbursement standards (Ref: High)					
Low	−0.50	0.11	−4.44	0.60 (0.48 to 0.75)	<0.001
Regional and population benefit package gap of medical insurance scheme (Ref: Unserious)					
Serious	−0.25	0.11	−2.16	0.78 (0.62 to 0.98)	0.030
Level of stakeholders’ discourse in medical insurance reform					
Scoring 1–10	0.19	0.03	6.98	1.21 (1.15 to 1.28)	<0.001
Fragmentation of the medical insurance system (Ref: No)					
Yes	−0.12	0.12	−1.06	0.88 (0.71 to 1.11)	0.288
Ability of vulnerable groups to participate in the insurance system (Ref: Sufficient)					
Insufficient	−0.24	0.11	−2.14	0.79 (0.63 to 0.98)	0.032
Degree of participation of medical insurance stakeholders in the reform (Ref: Low)					
High	0.65	0.12	5.30	1.91 (1.50 to 2.42)	<0.001
Medical insurance fund supervision (Ref: Ineffective)					
Effective	0.90	0.14	6.62	2.47 (1.89 to 3.22)	<0.001
Control of omissions from medical insurance coverage (Ref: Ineffective)					
Effective	1.40	0.13	10.44	4.04 (3.11 to 5.25)	<0.001
Management of online charity fundraising platforms (Ref: Ineffective)					
Effective	−0.03	0.12	−0.24	0.97 (0.78 to 1.22)	0.809

The regression equations have all controlled for significant confounding factors in the difference analysis.

### A possible non-linear relationship between the effectiveness of financial burden alleviation for people with catastrophic diseases and the level of stakeholders’ discourse

**Figure 1:**
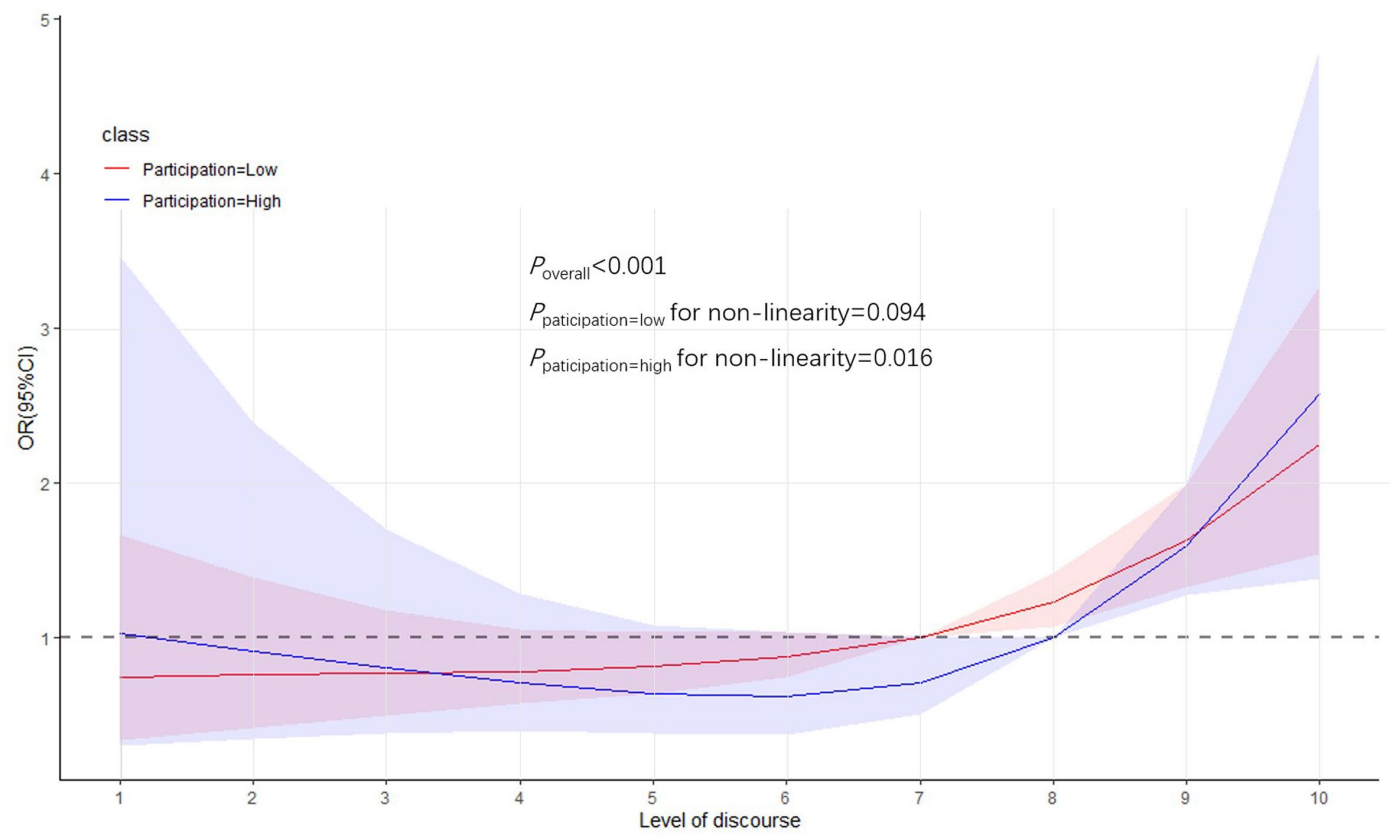
The graph illustrates the relationship between the level of discourse of medical insurance stakeholders and the evaluation on the effectiveness of financial burden alleviation for people with catastrophic diseases, showing specific trends in low and high levels of stakeholder involvement in medical insurance reform, respectively. The red line indicates the linear relationship between the two when stakeholders’ participation is low. The blue line indicates the non-linear relationship when stakeholders’ participation is high. The red and blue shades indicate the respective confidence intervals. The restricted cubic spline model adjusts for the influences of variables such as age, gender, education level, type of work unit, and medical insurance reimbursement standards, etc.

Flexible models using restricted cubic spline curved to visualize the relationship between the level of stakeholders’ discourse and the evaluation on the effectiveness of financial burden alleviation for people with catastrophic diseases. The results indicated a significant relationship between the two (*P*_overall_ < 0.001). Two scenarios were viewed. When the participation of medical insurance stakeholders in the reform is low, this relationship was linear (*P* for non-linearity = 0.094). That is, the likelihood of a positive evaluation of disease burden mitigation effects increased with increasing discourse degree scores. When stakeholder involvement in medical insurance reform is high, the relationship is similar to a flat “U” curve (*P* for non-linearity = 0.016). Further analysis of the high level of stakeholders’ participation. When the discourse degree score was less than “5″, the likelihood of a positive evaluation of disease burden relief decreased gradually with its increase; when the discourse degree score was greater than “7″, the likelihood of a positive evaluation of disease burden relief increased gradually with its increase. In particular, the likelihood of positive evaluation of disease burden relief tended to increase rapidly when the discourse degree score was greater than “8” Figure 1.

## Discussion

China has now basically achieved full coverage of basic medical insurance, and the level of medical protection for the whole society has been improved substantially. However, the phenomenon of a vicious circle between poverty and diseases still exists. This study investigated the evaluation of medical insurance-related professionals on the effect of financial burden relief for people with catastrophic diseases from a subjective perspective, which combined with their review and reflection on the possible impact of current medical insurance policies. We expect to explore the problems of the current medical insurance system in alleviating the financial burden of people with catastrophic diseases and to find strategies for governance. The following sections summarize the medical insurance-related factors found in this study that affect the financial burden of people with catastrophic diseases:

### The financial burden remains high for people with catastrophic diseases

In our survey, 50.92% of medical insurance professionals believed that the financial burden of people with catastrophic diseases has not yet been alleviated. China has continued to promote the reform of its medical insurance system and has made breakthroughs in recent years in tackling the problems of “difficult” and “expensive” access to medical care. However, China still faces disparities in the financial burden of medical care across regions and medical insurance systems, with relatively low protection against financial risk compared to developed countries (34). In Korea, around 20% of the population could incur a catastrophic financial burden (35). Catastrophic diseases were characterized by the high treatment costs and out-of-catalog expenses (36). Some patients with catastrophic diseases sometimes reflected a heavier burden. Data from The Lancet in 2011 showed that the incidence of CHE in China was 12.7%, which meant that 173 million people were in financial trouble paying for expensive medical treatment (37). In addition, in 2016, the figure was still 8.94% (38). Medical expenses due to illness were still a major cause of poverty for a large proportion of the population. Most families spent between 100,000 and 300,000 RMB on their children’s leukemia treatment. However, 63.03% of families could only get less than half of the reimbursement from medical insurance. This is related to the fact that 5.72% of respondents are uninsured, that there is a cap on reimbursement and that imported medicines are often used and not covered by medical insurance (39).

### Universal medical insurance coverage is critical

There are still some people with catastrophic diseases who are not covered by medical insurance (40). China has reached a milestone success in achieving the goal of universal health coverage. According to the Sixth National Medical Services Statistical Survey Report, China’s basic medical insurance coverage rate was about 96.8%, but there were still nearly 446 million uninsured people, and the rate increased by only 1.7 percentage points in 5 years compared with 2013. In some low-income countries, there remained significant barriers to achieving further increasing in coverage and providing essential financial protection to people with catastrophic diseases (3, 41). It should be noted that basic medical insurance alone cannot fundamentally address the financial burden of people with catastrophic diseases and that other supplementary medical insurance is needed to compensate for this. For example, major medical insurance and medical assistance (42–44). In contrast, supplemental medical insurance required a series of strict vetting processes and high admission criteria, and there were certain barriers for vulnerable people with catastrophic diseases to enter their coverage. Some studies have shown that major medical insurance has reduced the financial burden of patients with catastrophic diseases to some extent, and its coverage has been expanded compared to that of the basic medical insurance, but the coverage was still relatively small (20). Expanding medical insurance coverage coincided with Hallman’s proposed measures to address CHE and medical coverage for catastrophic diseases (45, 46).

### Insufficient medical insurance reimbursement standards

China’s basic medical insurance followed the principle of “wide population coverage and relatively low reimbursement level,” which mainly aimed at achieving rapid population coverage in the first phase of its development, but gradually led to the problem of unbalanced and insufficient development of the system. The inadequate design of the medical insurance system in terms of its financing, reimbursement levels, and relatively narrow benefit packages has led to a heavier financial burden for those vulnerable groups who suffer catastrophic diseases when medical insurance could not provide additional and preferential subsidies for vulnerable groups and effective compensation for catastrophic diseases (47). It has been reported that few households would incur CHE if out-of-pocket expenditure were reduced to 15% or less of total health expenditure (48). China has limited reimbursement for most people due to inconsistent levels of regional economic development (49–51).

### Differences in the benefit package of medical insurance scheme

It was unfair that each region set different limits and reimbursement rates, which often resulted in huge differences in insurance benefits between regions (52). Benefits in the eastern region of China were higher than in the central and western regions (51, 53). A study shows that the financial burden of healthcare in the south-eastern provinces is much lower than in other regions, allowing for better reimbursement of healthcare for local people (34).

### Governance-related measures to address above mentioned problems should be developed and implemented

Building a medical insurance governance system is necessary to reduce the financial burden of people with catastrophic diseases. This study was concerned with three main points. The first point is that governance is distinct from management, and the core of governance is “multiple participation” (54, 55). More medical insurance stakeholders involved in major reforms appeared to reduce the financial burden for the catastrophic disease population. The financial burden of catastrophic diseases was not only the cost of medical services but also the indirect financial burden such as lost wages, escort costs and transportation costs, as well as the hidden financial costs such as time and mental stress of the sick families. The medical insurance sector alone cannot achieve the goal of alleviating the financial burden of people with catastrophic diseases but requires multi-sectoral consultation and joint management (56, 57). The study has shown that geographically unstable and remote families, even if insured, tend to forgo medical care due to the time, difficulty, and cost associated with reaching a medical facility (58). This is where the transportation sector, medical institutions, and infrastructure sector should be involved in medical insurance efforts. The government of Taiwan province has led the way in this regard by coordinating organizations that assist people with rare diseases when they encounter problems with schooling, employment, or life support with the assistance of the Rare Diseases Foundation, schools, civic groups, and the mass media (23).

The second point is fund regulation. It is another aspect of governance. The World Bank’s Global Governance Indicators, published annually, cover the dimension of regulatory quality (59). Effective fund regulation can reduce the financial burden on people with catastrophic diseases. We suspect there are several reasons for this. Firstly it holds insurers accountable for their actions. Secondly it has been demonstrated that government regulation affects the length of stay of Medicare-covered patients (60) and that the presence or absence of medical insurance regulation makes a difference in the behavior of medical care providers in utilizing medical care resources (61). A unified approach to regulation is the best way to ensure fairness and efficiency in terms of financial solvency, consumer protection and equity (55).

The third point is more power to stakeholders outside of the government. Notably, the likelihood of financial burden relief for the catastrophic disease population increased by 18 percentage points for each unit increase in the level of medical insurance stakeholder involvement in reform discourse. Furthermore, in the grouped restricted cubic spline plot, the level of medical insurance stakeholder participation in reform discourse showed a relationship of positive contribution to the degree of financial burden relief for the population of the catastrophic disease, regardless of whether the level of major reform participation by medical insurance stakeholders was high or low. We believe that the distribution of discourse power is one of the key points of multi-subject cooperation, and the government should give more discourse power to each subject, while broadly mobilizing social forces to participate in the financial security of people with catastrophic diseases, so as to better play the function of the market. The government’s role gradually changed to that of rule maker, guide and regulator. As Sussex et al. suggested, the government should actively guide social forces to participate in the livelihood security of the sick through the timely introduction of relevant policies, and encourage social charity forces to play a greater role in the field of medical assistance (62). It has been demonstrated that there are rapidly growing policy-making advantages that result from an equal partnership between the government and social welfare organizations in the field of medical charity (63). Government should intervene moderately in the insurance market by finding a new mix of insurance market roles and government roles (64). Overall, the decentralization of government is likely to positively affect the relationship where medical insurance alleviates the financial burden of people with catastrophic diseases.

## Limitations

This paper was based on self-reported subjective perception data and cognitive information based on a certain recall period. Therefore, the judgments of the relevant entries may be inaccurate due to recall bias. Our sample may not be nationally representative, and caution should be exercised when quoting facts that apply to the whole country. In addition, our findings were related to personal affective experiences, and caution was needed in generalizing the findings to the same meaning of financial burden, etc., as indicated by objective indicators.

## Conclusion

China’s current medical insurance policies have not yet fully addressed the needs of vulnerable populations, and multidimensional health risks add to the financial burden of people with catastrophic diseases. China still faces challenges such as relatively insufficient coverage for vulnerable populations, inadequate depth of coverage, and the gap in treatment differences between different systems and regions. To further alleviate the financial burden of people with catastrophic diseases and improve China’s medical insurance governance capacity, the government should encourage the participation of multiple subjects in the significant reform process of medical insurance. Then increase the level of discourse on the market and social subjects, and give a certain degree of equal consultation rights, while government departments should act more as rule-makers and regulators to control the overall situation.

## Data availability statement

The raw data supporting the conclusions of this article will be made available by the authors, without undue reservation.

## Author contributions

PG and RW drafted the manuscript. YQ performed the data processing and analysis. LS guided the manuscript framework. JLi and JLiu performed the image drawing and preliminary review. KW, HS, and HL performed the literature search. YL, YH, YC, and ZK performed quality control and review of the manuscript. QW revised the manuscript and gave critical suggestions. All authors contributed to the article and approved the submitted version.

## Funding

This work was supported by the National Social Science Foundation of China (Grant No.19AZD013).

## Conflict of interest

The authors declare that the research was conducted in the absence of any commercial or financial relationships that could be construed as a potential conflict of interest.

## Publisher’s note

All claims expressed in this article are solely those of the authors and do not necessarily represent those of their affiliated organizations, or those of the publisher, the editors and the reviewers. Any product that may be evaluated in this article, or claim that may be made by its manufacturer, is not guaranteed or endorsed by the publisher.
